# Moyamoya disease and syndrome: a review

**DOI:** 10.1590/0100-3984.2021.0010

**Published:** 2022

**Authors:** Zeferino Demartini Jr., Bernardo CA. Teixeira, Gelson Luis Koppe, Luana A. Maranha Gatto, Alex Roman, Renato Puppi Munhoz

**Affiliations:** Complexo Hospital de Clínicas - Universidade Federal do Paraná (UFPR), Curitiba, PR, Brazil.; Complexo Hospital Pequeno Príncipe, Curitiba. PR, Brazil.; Hospital Universitário Cajuru - Pontifícia Universidade Católica do Paraná (PUCPR), Curitiba, PR, Brazil.; Cleveland Clinic, Abu-Dhabi, United Arab Emirates.; Toronto Western Hospital, Division of Neurology, University of Toronto, Toronto, Canada.

**Keywords:** Moyamoya disease, Cerebrovascular disorders, Intracranial arterial diseases, Cerebral arterial diseases, Cerebral revas-cularization, Doença de moyamoya, Transtornos cerebrovasculares, Acidente vascular cerebral, Doenças arteriais intracranianas, Doenças arteriais cerebrais, Revascularização cerebral

## Abstract

Moyamoya disease is a chronic occlusive cerebrovascular disease that is non-inflammatory and non-atherosclerotic. It is characterized by endothelial hyperplasia and fibrosis of the intracranial portion of the carotid artery and its proximal branches, leading to progressive stenosis and occlusion, often clinically manifesting as ischemic or hemorrhagic stroke with high rates of morbidity and mortality. On cerebral angiography, the formation of collateral vessels has the appearance of a puff of smoke (moyamoya in Japanese), which became more conspicuous with the refinement of modern imaging techniques. When there is associated disease, it is known as moyamoya syndrome. Treatments are currently limited, although surgical revascularization may prevent ischemic events and preserve quality of life. In this review, we summarize recent advances in moyamoya disease, covering aspects of epidemiology, etiology, presentation, imaging, and treatment strategies.

## Abstract (pt)

A doença de moyamoya, ou doença cerebrovascular oclusiva crônica, é uma afecção não inflamatória e não aterosclerótica, caracterizada por hiperplasia endotelial e fibrose dos segmentos intracranianos das artérias carótidas internas e da porção proximal de seus ramos. Isso provoca estenose progressiva e oclusão, frequentemente manifestada clinicamente como isquemia cerebral ou hemorragia intracraniana, com alta morbimortalidade. A formação compensatória de vasos colaterais produz, na angiografia encefálica, um aspecto de nuvem de fumaça (moyamoya, em japonês). Quando existe doença subjacente que possa estar relacionada, a doença recebe o nome de síndrome de moyamoya. Embora a incidência esteja aumentando graças aos novos métodos diagnósticos, as estratégias terapêuticas ainda são limitadas. O diagnóstico precoce permite cirurgias de revascularização cerebral que podem evitar novos acidentes vasculares e melhorar a qualidade de vida. Nesta revisão são apresentados os avanços recentes sobre a doença de moyamoya, citando aspectos de epidemiologia, etiologia, apresentação, exames diagnósticos e tratamento.

## INTRODUCTION

Takeuchi et al.^(1)^ first described the so-called “hypoplasia of bilateral internal carotid arteries” in a paper published in 1957. The disorder was later classified as acquired and progressive, and it was not until 1969 that Suzuki et al.^(2)^ introduced the term “moyamoya” (Japanese word for “hazy”) as an allusion to the similarity that the cerebral angiographic findings, specifically the groups of vessels sprouting to compensate for the progressive stenosis, had to a puff of smoke.

Moyamoya disease (MMD), a chronic occlusive cere-brovascular disease, is a non-atherosclerotic structural arterial abnormality characterized by progressive stenosis or occlusion of the intracranial internal carotid arteries (ICAs) and their proximal branches, with abnormal formation of collateral vessels, known as rete mirabile(^3^,^4^,^5^,^6^,^7^). The steno-occlusive changes in MMD are usually bilateral, although unilateral involvement does not exclude the diagnosis^(5)^. The main pathophysiology of MMD is thought to be chronic brain hypoperfusion as a result of steno-occlusive changes around the ICA bifurcation(^4^,^6^). Unlike what is seen in conditions such as M1 stenosis, in which the lesions affect only one vessel, the hypoperfusion in MMD involves adjacent vascular territories, causing more significant ischemia^(8)^, which chronically induces the formation of collateral vessels of arterial branches of the ICA (prior to its bifurcation) and of the posterior circulation^(6)^. The collaterals arise mainly from the choroidal arteries (including the anterior, posterolateral, and posteromedial choroidal arteries), although transcranial and transdural collaterals are also commonly seen in the external carotid arteries, the ophthalmic artery, and the meningeal artery^(6)^. As the disease progresses, the volume of blood flow through the collateral channels increases, which may lead to excessive hemodynamic stress, causing rupture and intracranial hemorrhage^(7)^.

Patients with a vasculopathy similar to MMD in the setting of another (underlying) disease are classified as having moyamoya syndrome^(9)^. Such underlying diseases include Down syndrome, cranial irradiation, sickle cell disease, neurofibromatosis type 1, and thyroid disease, as well as, less frequently, systemic lupus erythematous, Turner syndrome, and Noonan syndrome^(6)^. In Western countries, moyamoya syndrome occurs in less than 20% of diagnosed cases of MMD, whereas it accounts for 34–60% of cases of moyamoya-like vasculopathy in children^(10)^.

Although the pathogenesis of MMD is still not fully understood and the available treatments are rather limited, producing unfavorable outcomes, there have been recent advances that are promising^(5)^. Here, we review recent developments in clinical and imaging findings, as well as the treatment options.

## EPIDEMIOLOGY

There have been documented cases of MMD all over the world, and it is the most common form of pediatric cerebrovascular disease in East Asian countries^(11)^. The prevalence and incidence of the disease are higher among Asian individuals, especially those of Japanese, Korean, or Chinese descent(^5^,^6^). In recent decades, the incidence and prevalence of MMD have increased progressively and its prevalence among males has come to equal or slightly surpass that among females^(5)^. The change in prevalence is seen as a consequence of better diagnostic accuracy due to modern, noninvasive radiological techniques, as well as to increased patient longevity after treatment with advanced therapeutic strategies^(5)^. Studies have also found that there are two main age ranges for the onset of MMD: 5–10 years and 25–49 years(^12^,^13^). The prevalence of symptomatic MMD in the United States showed a fourfold increase between 2005 and 2008, 32 years being the median age of symptom onset and there being a trend toward a predominance of females^(14)^.

## ETIOLOGY AND HISTOLOGICAL FINDINGS

Despite many advances, the exact pathophysiological triggers and precise timeframe of the progression of MMD remain unknown, although lines of evidence have indicated pathogenic pathways as diverse as those of angiogenesis, genetics, the immune system, and inflammation^(5)^. The pathogenesis of MMD has been associated with several angiogenesis-related factors, such as endothelial colonyforming cells and cytokines, including vascular endothelial growth factor, transforming growth factor beta 1, basic fibroblast growth factor, and hepatocyte growth factor(^5^,^15^). It has also been suggested that mitochondrial abnormalities and CD34-positive cells, as well as mRNA and protein expression of elastin, play a role in the occurrence and development of MMD(^5^,^15^).

A genetic contribution is suspected on the basis of the familial cases observed and the aforementioned strong link with ethnicity^(5)^. In East Asian populations, the ring finger protein 213 on 17q25.3 is considered to be a susceptibility gene for MMD^(16)^, ring finger protein 213 variant p.R4810K being strongly associated with familial MMD in Japan, China and Korea^(17)^. In addition, autoimmune responses have been implicated as the underlying mechanism of MMD^(5)^. Plasma inflammatory factors, including interleukin-1 beta, monocyte chemoattractant protein-1, and stromal cell-derived factor-1 alpha, have been found to be elevated in patients with MMD, suggesting immunemediated inflammation as a contributing factor in this equation^(18)^. Furthermore, the role of immunological and inflammatory mediators in the pathogenesis of MMD includes abnormal IgG deposition into elastic layers and infiltration by T cells, macrophages, and S100A4-positive smooth muscle cells in the intimal layer^(19)^.

From a histological standpoint, the findings of MMD do not seem to differ between Asians and individuals of other ethnicities. Such findings include thickening and undulation of the internal elastic lamina, as well as fibrocellular hypertrophy and proliferation of smooth muscle cells in the tunica intima, affecting the distal parts of the ICAs and proximal segments of the anterior and middle cerebral arteries. Thinning of the tunica media is a late finding that tends to be more pronounced among adult patients with MMD than among pediatric patients with the disease^(20)^. Collateral vessels may show fragmented internal elastic lamina and similar thinning of the media with isolated microaneurysms^(21)^.

## CLINICAL PRESENTATION

The onset of MMD may occur at any age, from childhood to adulthood, the most common symptoms being cerebral ischemia and intracranial hemorrhage. The initial manifestations include transient ischemic attack (TIA), ischemic stroke, hemorrhage, headache, seizures, cognitive impairment, and movement disorders^(5)^. Although ischemic and hemorrhagic events are the most common presentations, their frequencies differ between pediatric and adult patients. In childhood and adolescence, ischemia is the most common presentation (occurring in 73.9–97.5% of cases), whereas hemorrhage is much less common (occurring in only 2.5–8.0%). In adults with MMD(^6^,^13^), there is a higher prevalence of intracranial bleeding (19.1–42.3%), with relatively less TIA or cerebral infarction (57.7–70.0%). Variations in age, territory of vascular involvement, stenosis severity, and brain territories affected account for a wide range of clinical manifestations and timeframes(^3^,^5^). Typically, the progression of arterial stenosis leads to cerebral hypoperfusion, causing multiple and recurrent ischemic events^(5)^. Cerebral infarction is a common presentation in adults, whereas TIA is more common in children and adolescents^(22)^. In pediatric patients with MMD, TIA episodes frequently occur during hyperventilation due to crying or are caused by fever or dehydration^(5)^. These triggers may induce vasoconstriction or an overall decrease in cerebral blood flow (CBF), which is compounded by the fact that cerebral metabolic demand is much higher in the first decade of life, decreasing thereafter^(22)^. The risk of symptomatic MMD recurrence is 18% in the first year after the initial presentation and increases by 5% per year after that, with a 5-year cumulative risk of approximately 40%, and lacunar infarcts present better functional outcomes after revascularization^(23)^. However, ischemic events affecting the posterior circulation, including the territories of the vertebral artery, basilar artery, and posterior cerebral artery (PCA), may worsen the clinical course and outcomes^(5)^. In adults with MMD, the white matter is more susceptible to injury than is the gray matter, such injury leading to cognitive dysfunction^(5)^. Cerebral hemorrhage is more common in adults with MMD, often with outcomes worse than those seen in children and adolescents^(5)^. Cerebral hemorrhage typically occurs due to rupture of friable collateral arteries, frequently in intraventricular and lobar areas, because most of the collateral vessels arise from the choroidal system, which has been associated with aneurysms^(6)^. The posterior communicating arteries can also be a source of bleeding, and the presence of multiple microbleeds predicts the future occurrence of more clinically significant hemorrhage^(24)^.

The concept of unstable MMD, defined as cases of rapid progression or recurrent stroke, represents a more clinically challenging condition, most often seen in patients under three years of age and in those with an underlying disorder. Because unstable MMD represents a potential risk factor for perioperative ischemic complications, its early recognition may create a valuable window of opportunity for perioperative-focused management and surgical risk stratification, thus improving surgical out-comes^(7)^. Movement disorders occur in 3–4% of children with MMD, occurring even less frequently among adults; the most common forms are hemichorea-hemiballismus or chorea-ballismus, followed by dystonia (segmental, generalized, hemidystonia, or acute status dystonicus) and, less frequently, ataxia, myoclonus, and isolated limbshaking^(7)^. Any of those may occur in combination, and each may manifest as a continuous, paroxysmal, or hyper-ventilation-induced phenomenon^(25)^.

## DIAGNOSTIC TESTS

**Figure 1:**
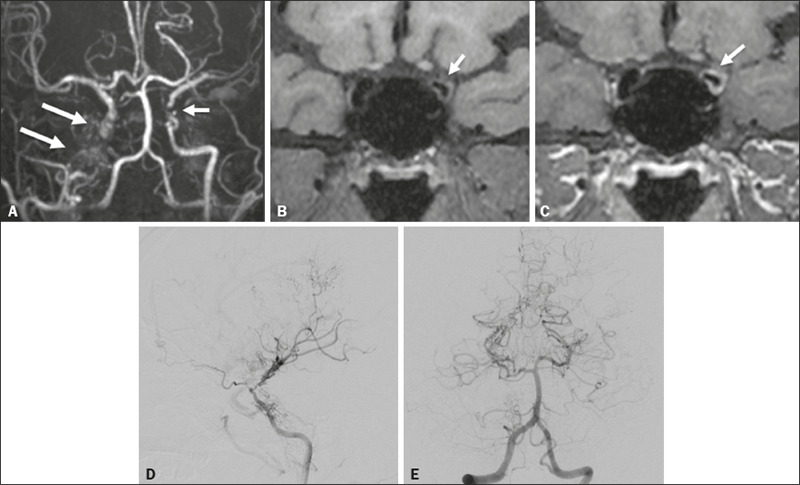
An 11-year-old girl with a learning disorder who developed right hemichorea. **A:** MRI angiography showing stenosis of the left ICA (short arrow) and collateral circulation to the right hemisphere (long arrows). Vessel wall imaging before and after contrast administration (**B** and **C**, respectively) showing diffuse thickening and enhancement at the site of the left ICA stenosis. Selective DSA of the left ICA (**D**) and right vertebral artery (**E**) showing carotid stenosis, whereas collaterals vessels have the moyamoya (“puff of smoke”) aspect.

Although initial investigation with computed tomography (CT) and magnetic resonance imaging (MRI) typically show hemorrhage in adults and ischemic lesion in children (Figure 1), failure to perform specific vascular studies may result in misdiagnosis^(5)^. The diagnosis of MMD is based on anatomical and functional aspects^(5)^. First-line imaging examinations include CT angiography and MRI angiography to identify anatomical characteristics, such as steno-occlusive changes and the formation of collateral vessels^(5)^, as well as digital subtraction angiography (DSA), which continues to be the gold standard examination to confirm the diagnosis and staging^(26)^. In fact, the definitive diagnosis of MMD is still based on the brain DSA findings, which define the dynamic vascular changes and allow the disease to be staged with systems such as the Suzuki classification(^2^,^5^), as summarized in Table 1. The application of this technique and classification is also useful for the definition of risks. For instance, when the DSA findings of ischemic MMD are compared with those of hemorrhagic MMD, the latter generally presents with a Suzuki stage that is more advanced, more intracranial aneurysms, development of fetal-type PCA, and collateral flow including ethmoidal and transdural vessels^(4)^. Anterior carotid artery occlusion occurs more frequently in patients with intraventricular or deep intraparenchymal hemorrhage than in those with lobar hemorrhage, suggesting that the underlying cause of the elevated risk for these complications in MMD is major artery occlusion compensated by collateral vessels^(4)^. A study comparing 7.0-T and 3.0-T MRI in patients with MMD detected no statistically significant differences in ICA diameter, stage, or ivy sign score^(27)^. The functional component is assessed using techniques such as perfusion MRI, single-photon emission CT, positron emission tomography, perfusion CT, and xenon-enhanced perfusion CT, aiming to quantify CBF and cerebrovascular reserve capacity(^5^,^27^).

**Table:** Angiographic stages of MMD, as proposed by Suzuki et al.^(2)^.

Stage	Angiographic findings
1	Narrowing begins at the ICA bifurcation
2	Moyamoya collaterals seen around narrowed vessels
3	Worsening of collateral vessels
4	Exacerbation of narrowed vessels and initial weakening of the collaterals
5	Occlusion of large associated vessels and more pronounced reduction of surrounding moyamoya changes
6	Disappearance of the moyamoya collaterals and vessels of the ICA system, which have come to be supplied by the external carotid artery

**Figure 2:**
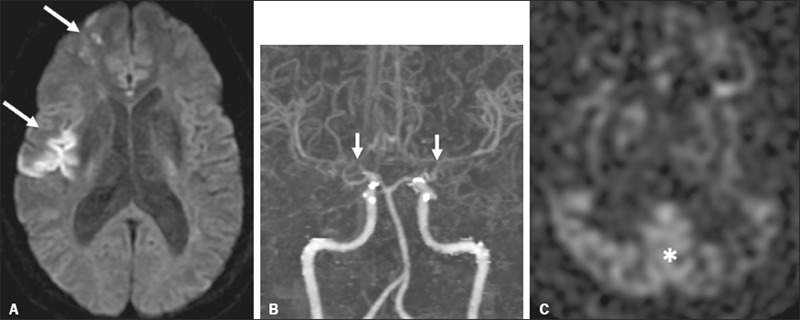
A 46-year-old male presenting with motor deficit and TIAs. **A:** Diffusion-weighted MRI showing infarcts (arrows) in the territory of right middle cerebral artery and watershed area. **B:** Three-dimensional CT angiography showing bilateral stenosis of the terminal carotid arteries (arrows) and reduced opacification of the middle cerebral arteries. **C:** Arterial spin-labeling perfusion MRI showing extensive reductions in blood flow in the territories of the anterior and middle cerebral arteries, with preserved posterior circulation (asterisk).

The development of new imaging modalities has improved the diagnosis, longitudinal monitoring, and postoperative follow-up of MMD. For instance, dynamic susceptibility-weighted contrast-enhanced MRI is a safe and useful technique to show the passage of a gadolinium-based contrast agent in order to measure brain perfusion and thus measure cerebrovascular reserve capacity in patients with MMD^(28)^. Blood oxygen level-dependent functional MRI, typically employed to observe areas of brain activation, is also useful in MMD due to its ability to assess hemodynamic changes^(29)^. In addition, resting-state functional MRI can detect spontaneous fluctuations of blood oxygen level-dependent signals over time in patients with MMD, suggesting that it is a more sophisticated method to evaluate the functional organization of the brain^(30)^. Other advanced MRI techniques, such as high-resolution MRI and high-resolution vessel wall imaging, may allow early detection of vascular changes, as well as facilitating the differential diagnosis of various causes of arterial stenosis^(31)^. In patients with MMD, high-resolution MRI may reveal the absence of a hyperintense juxtaluminal band in T2-weighted sequences, narrowing of the middle cerebral artery, and mild homogeneous concentric wall enhancement in the distal ICA, unlike the eccentric wall thickening seen in patients with atherosclerotic plaques^(31)^. Highresolution MRI may also identify additional intracranial atherosclerotic plaques in the assessment of prognosis in adult patients with combined MMD and atherogenic risk factors^(5)^. Finally, arterial spin-labeling is an MRI perfusion technique that does not involve the use of contrast media and allows qualitative and quantitative analyses of CBF through the use of labeled (tagged) water molecules in the arterial blood. It has been shown to identify and determine the intensity of collateral flow in patients with MMD, at baseline^(32)^ and after revascularization procedures^(33)^, as illustrated in Figure 2. In addition to brain imaging, diagnostic tests for the investigation of associated conditions are essential in patients with MMD^(10)^.

## TREATMENT

Although there is currently no specific therapeutic strategy that is effective in preventing or reversing the background vascular abnormalities in MMD, interventions used for stroke prophylaxis have probably changed the natural history of the disease^(5)^. Treatments can be classified as conservative or interventional, with the caveat that there is as yet no data on long-term treatment comparing conservative and surgical management of MMD^(5)^.

The cornerstones of the clinical approach to MMD are prophylactic and generic symptomatic treatment, such as the use of antiplatelet drugs, anticonvulsant drugs, and pain management^(5)^. Treatment with acetylsalicylic acid is strongly recommended to prevent recurrence of ischemic attacks, and clopidogrel or another thienopyridine may be used when acetylsalicylic acid is not tolerated or is ineffective^(5)^. Therefore, pharmacological treatment is directed at aggressive prevention of new neurovascular events and no single-drug regimen is accepted as a gold standard for ischemic or hemorrhagic complications. In addition, rigid control of additional risk factors, such as dyslipidemia, hypertension, and diabetes, is highly recommended(^3^,^5^).

The main goal of surgical treatment is to minimize cerebral ischemia by enhancing CBF and decreasing the hemodynamic stress that causes cerebral hemorrhage^(5)^. Revascularization surgery prevents stroke and secondary hemorrhage in patients with MMD, lowering the rate of recurrence of ischemic attacks and producing clinical outcomes that are significantly more favorable than are those achieved with conservative treatment(^5^,^6^,^10^). Surgical revascularization employs external carotid artery branches such as the superficial temporal artery and occipital artery as donor arteries, and it can be divided into direct and indirect types. A few direct bypass techniques have been tested, including superficial temporal artery-middle cerebral artery anastomosis^(34)^, superficial temporal artery-anterior cerebral artery anastomosis^(35)^, and occipital artery-PCA anastomosis^(36)^. Direct anastomosis leads to an increase in immediate cerebral perfusion and should therefore be considered the first-line treatment^(6)^. When that is not possible, indirect methods are useful and technically easier to perform, although they require more time to adequately restore blood flow^(6)^. The main indirect revascularization technique is synangiosis and aims the development of collateral circulation toward the brain cortex using connective tissues such as the scalp, muscle, and dura mater^(6)^. Several indirect “onlay” techniques have been described: encephalo-duro-arterio-synangiosis^(37)^; encephalo-myo-synangiosis^(38)^; and en-cephalo-duro-arterio-myo-synangiosis^(39)^. There are also combinations of direct and indirect bypass^(34)^, as well as multiple burr hole surgery^(40)^. A meta-analysis showed that, in adults with symptomatic MMD, surgery is superior to conservative treatment in terms of preventing future strokes, and direct bypass seems to be more effective than is indirect bypass, producing favorable long-term results^(41)^. In another meta-analysis of pediatric MMD, all revascularization options were found to be effective, although the incidence of postoperative stroke was 1 episode/190.3 patient-years after direct bypass alone, 1 episode/108.9 patient-years after combined bypass, and 1 episode/61.1 patient-years after indirect bypass alone^(42)^. The true benefit of revascularization for adults with asymptomatic MMD remains unknown, and there is as yet no consensus regarding the need for or timing of surgical intervention in such patients(^5^,^6^). For patients with MMD in an advanced Suzuki stage, indirect revascularization has been shown to improve in cerebral perfusion, although not to reduce the risk of stroke recurrence compared with conservative treatment^(43)^. In addition, endovascular obliteration of pseudoaneurysms in collaterals vessels is emerging as an option to prevent recurrent bleeding in patients with hemorrhagic MMD^(6)^. However, because parent arteries are functional channels, their irrigated territory must be carefully evaluated during microcatheterization to assess the risk and benefits of such procedures^(6)^.

**Figure 3:**
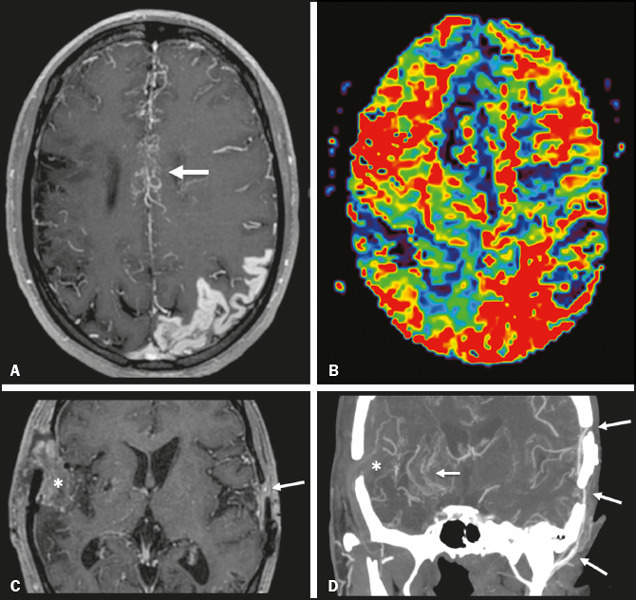
A 22-year-old female with chronic headache, together with incipient seizures and MMD. **A:** Contrast-enhanced MRI showing chronic right frontotemporal infarct and subacute left parietotemporal infarct, as well as collateral circulation surrounding the middle and anterior cerebral arteries (arrow). **B:** Dynamic susceptibility contrast perfusion MRI showing increased time-to-maximum values in both cerebral hemispheres. Axial contrast-enhanced MRI (**C**) and coronal CT angiography (**D**) after right-side encephalo-duro-arterio-synangiosis and left-side bypass between the superficial temporal and middle cerebral arteries (long arrows) showing moyamoya collaterals (short arrow) and signs of revascularization (asterisks).

After surgical intervention, long-term clinical and imaging follow-up is required to ensure the effectiveness of the procedures (Figure 3). Postoperative collateral formation may be evaluated by cerebral angiography and classified in accordance with the Matsushima grading scale^(44)^, as shown in Table 2.

**Table:** Postoperative angiographic grading of collaterals from the external carotid artery, as proposed by Matsushima et al.^(44)^.

Grade	Angiographic findings
A	> 2/3 of the middle cerebral artery territory perfused by the synangiosis
B	2/3 to 1/3 of the middle cerebral artery territory perfused by the synangiosis
C	< 1/3 of the middle cerebral artery territory perfused by the synangiosis

## CONCLUSION

Advances in diagnostic methods have led to improved diagnostic sensitivity and specificity, enabling early treatment of MMD. A better understanding of the patho-physiology and clinical course of the disease, as well as of the most effective therapeutic approaches, is critical to achieving better outcomes.
